# Screening and In Silico Analyses of the Yeast *Saccharomyces cerevisiae* Σ1278b Bank Mutants Using Citral as a Natural Antimicrobial

**DOI:** 10.3390/foods13101457

**Published:** 2024-05-08

**Authors:** Rolla El Harati, Francesco Fancello, Chiara Multineddu, Giacomo Zara, Severino Zara

**Affiliations:** Department di Agricultural Sciences, University of Sassari, 07100 Sassari, Italy; r.elharati@studenti.uniss.it (R.E.H.); fancello@uniss.it (F.F.); chiaramultineddu2@gmail.com (C.M.); gzara@uniss.it (G.Z.)

**Keywords:** reactive oxygen species, antimicrobial function, *S. cerevisiae* gene bank, antimicrobials, deletome

## Abstract

The antimicrobial function of citral, one of the main compounds of the essential oils (EO) of the *Citrus* genus, and widely used by the food industry toward spoilage yeast, was previously proven. In this study, the possible mode of action of citral against yeast cells was evaluated by using a global deletome approach. Firstly, the suitability of *Saccharomyces cerevisiae* Σ1278b to serve as model yeast was assessed by determining its sensitivity to citral (MIC = 0.5 μL/mL). Subsequently, the complete library of Σ1278b haploid mutants deleted in 4019 non-essential genes was screened to identify potential molecular targets of citral. Finally, the deleted genes in the 590 mutants showing increased citral resistance was analyzed with an in-silico approach (Gene Ontology). The significantly enriched GO Terms were “cytoplasm”, “vacuole”, and “mitochondrion” (cellular components); “catalytic activity” (molecular function); “pseudohyphal growth” (biological process). For molecular function, resistant mutants were grouped into thiosulfate sulfur transferase activity, transferase activity, and oxidoreductase activity; for cellular components, resistant mutants were grouped as: cytoplasm, intracellular organelle, membrane-bounded organelle, mitochondrion, organelle membrane, and vacuole; and finally, with regard to biological process, deleted genes were grouped as: pseudohyphal growth, mitochondrion organization, lipid metabolic process, DNA recombination and repair, and proteolysis. Interestingly, many identified genes were associated with the cellular response to oxidative stress and ROS scavenging. These findings have important implications for the development of citral-based antimicrobials and the elucidation of its mechanism of action.

## 1. Introduction

*Saccharomyces cerevisiae* can endure a reversible developmental transition from a single-cell budding yeast into a multicellular filamentous form [1]. This dimorphic shift facilitates haploid cells to form biofilms on a semisolid medium [2] and to penetrate agar in response to carbon deprivation (haploid invasive growth) [3,4], whereas it enables diploid cells to form a bundle of elongated cells known as pseudohyphae in response to nitrogen starvation [5]. In the food realm, biofilm formation reduces the quality of dairy products, juices production lines, drinking water systems, beer and wine [6]. It has also been shown that the yeast strain Σ 1278b naturally forms biofilm in liquid medium on solid surfaces such as polystyrenes caused by the expression of the protein Flo11p [2,7,8,9,10].

We are currently facing an increase in the resistance to antifungal molecules, affecting the number of compounds available to control spoilage and pathogenic microorganisms, and producing a need to find new alternatives. Plant extracts contain molecules with antimicrobial activity, among which essential oils offer an alternative to “synthetic agents” [11]. These natural compounds are defined to be “generally regarded as safe” (GRAS) by the US Food and Drug Administration and offer promising treatments in medicine and applications in the cosmetic, pharmaceutical and food industries [12,13,14,15,16]. Essential oils are known to show biological activity, like antimicrobial and antioxidant activity. They are characterized as complex mixtures of natural compounds [17]. In recent years, the antifungal activity of citrus essential oil has been the object of several studies highlighting its use against *Aspergillus* and *Penicillium* species [18].

**Figure 1:**
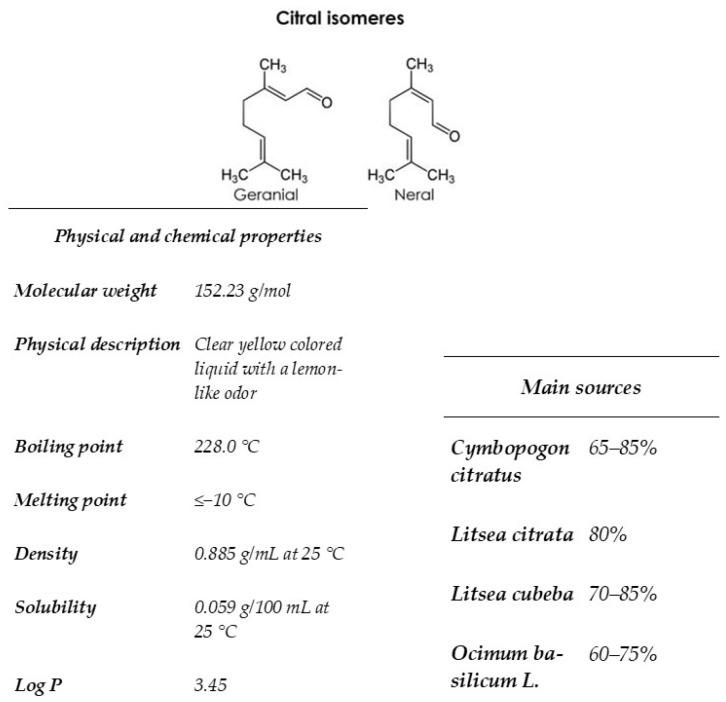
Reprinted from “Gutiérrez-Pacheco, M.M.; Torres-Moreno, H.; Flores-Lopez, M.L.; Velázquez Guadarrama, N.; Ayala-Zavala, J.F.; Ortega-Ramírez, L.A.; López-Romero, J.C (2023). Chemical structures, main sources, and citral’s physical and chemical properties. MDPI. Copyright 2023” [32]. Citral is an acyclic monoterpenoid aldehyde composed of two geometrical isomers, geranial and neral. This monoterpene is present in many plants, citrus fruits, herbs, and grasses. It is very volatile, exhibits lipophilic activity, and has low solubility in water.

Citral is found in many species of aromatic plants such as lemon myrtle, lemongrass, lemon, orange. This terpenoide is composed of two aldehydes stereoisomers geranial (trans-citral) and neral (cis-citral) [19]. This component is highly volatile, exhibits a lipophilic action, and presents low solubility in water (Figure 1). Much in vitro research has described the biological activity of citral, including antibacterial activity [20,21], anti-inflammatory activity [22], and antifungal effects [23,24,25,26]. Mitropoulou et al. [27] showed that citral interacts with membrane fatty acids of eukaryotic cells such as *Geotrichum citri-aurantii*. In addition, this component induces mitochondrial morphology, reduces intracellular ATP, and prohibits several enzymes of the tricarboxylic acid pathway of *Penicillium digitatum*) [18]. Bard et al. [28] evaluated the antimicrobial activity of geranial and neral (the two isomers of citral) against *S. cerevisiae*, which have previously been used as a model system in food spoilage. The environmental stress tolerance mechanism of yeast is based primarily on two factors: cell resistance to environmental stresses and cell self-repair after damage [29]. Since oxidative stress primarily causes the accumulation of harmful reactive oxygen species (ROS), the source of ROS and cell response have received considerable attention [30]. Shi et al. [20] showed that citral increased the intracellular reactive oxygen species (ROS) allied with alterations in mitochondrial activity, and caused apoptosis cell death by the metacaspase-dependent pathway. In the same study, cell membrane dysfunction was related to the synergy of terpenoids with the lipids of the membrane bilayer [28], inducing leakage of potassium and cell constituents [28,31]. Fancello et al. [17] have shown the antifungal effect of Pompia leaf EO on *Saccharomyces cerevisiae* (E1118) with a MIC of 0.5 µL/mL, and this activity is due to citral, its main component. 

Greater consumer awareness and concern regarding synthetic chemical additives have led different types of industries to control microbial spoilage and hazards using natural preservatives, such as plant essential oils and their components that possess antimicrobial activity. So far, there has been inadequate attention given to the ability of microorganisms to develop resistance to these antibiotic alternatives and limited studies have been conducted to evaluate microbial adaptive response and virulence changes toward sublethal natural compounds exposure. Therefore, new phenotypic and genetic studies are necessary to understand the mechanism of action of natural preservatives to evaluate the possible development of microbial tolerance or resistance to natural compounds.

In this study, we focused on the screening of the bank of mutants of *S. cerevisiae* Σ1278b for the anti-microbial function of citral. The bank of mutants of *S. cerevisiae* Σ1278b was created by PCR amplification of the corresponding deletion alleles, from the S288c reference strain collection [33,34]. The diploid Σ1278b strain was engineered by holding synthetic genetic array (SGA) mating-type specific reporters [35,36]. The library contains about 4019 haploids that each harbor a single deletion in a non-essential gene [37]. Thus, the molecular mechanism of the deleted genes in mutants that are able to resist citral helps to understand the antifungal mechanisms of citral. In addition, by an in silico analysis we developed hypotheses that relate the function of the known genes to citral-mediated anti-microbial activity. These efforts contribute new knowledge that will advance our long-term goal of determining the mechanism of action of these molecules, to develop effective methods for preventing unwanted microbial growth, and to deepen our knowledge about the tolerance or resistance of microorganisms to natural compounds.

## 2. Materials and Methods

### 2.1. S. cerevisiae Σ1278b Bank of Mutants

*S. cerevisiae* Σ1278b haploids were constructed by switching each open reading frame (ORF) in Σ1278b (*MAT*a can1D::STE2p-spHIS5 lyp1D:: STE3p-LEU2 his3::HisG leu2D ura3D) with a kanMX cassette received from the S288c deletion collection [37]. Wild Type was used as a reference strain and mutants’ alleles were similar to the S288c collection [34]. The library consisted of 4019 haploids that each harbor a single deletion in a non-essential gene screened in the Σ1278b strain. The library was replicated in liquid YEPD (Yeast Extract, Peptone, Dextrose) + G418 Sulfate (Gold Biotechnology, St. Louis, MO, USA) + 20% glycerol and stored at −80 °C. The library is not distributed commercially but has been made available to the Department of Agricultural Sciences, Section of Microbiology, (University of Sassari) by Boone Lab, University of Toronto, Canada [38].

### 2.2. Determination of the MIC of Citral on S. cerevisiae Σ1278b

The minimal inhibitory concentration (MIC) was determined as the lowest concentration of citral (3,7-dimethyl-2,6-octadienal, geranial and neral mixture), (Sigma Aldrich, St. Louis, MO, USA), inhibiting visible growth of the tested microorganism. Citral stock solution was first prepared with a concentration of 40 μL/mL. Stock solution was then diluted with sterile water and 1% DMSO, giving a succession of concentrations ranging from 0.078 to 20 μL/mL [17]. Overnight culture of the wild-type yeast *S. cerevisiae* Σ1278b was used to prepare the microbial inoculation used for the test, reaching an OD of 0.2. Aliquots of 100 μL of diluted inoculation at desired cell concentration were added to each well in the 96-well micro-dilution polystyrene plate, which already contained 100 μL of the citral dilution. The plates were then incubated at 30 °C for 24 h. DMSO alone (at 1% concentration) and sterile water was used as positive control. Each experiment was repeated twice. The preculture and dilutions of the parental strain were prepared with an inoculum solution of YEPD (2% bacto-peptone, 1% yeast extract, and 2% glucose) reaching an OD of circa 0.2 (about 150 μL of the preculture for 10 mL of the prepared medium) in YEPD at 30 °C for 24 h.

### 2.3. Screening of the S. cerevisiae Σ1278b Bank of Mutants

**Figure 2:**
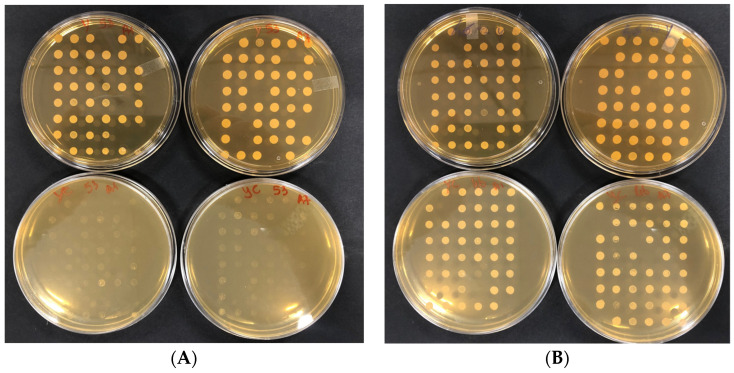
Representative plates showing the citral-related phenotype on solid medium. Mutants were grown on liquid YEPD+G418 in 96 bottom-well plates and replicated on YEPD+G418 (plates up) and on YEPD+G418+citral (plates down). (**A**) sensitive mutants. (**B**) resistant mutants.

Mutants were prepared by growing them individually overnight in rich medium YEPD broth + G418 Sulfate, in 96-well plates for minimizing population bias due to differences in growth fitness that in bulk culture would place slower growing clones at a disadvantage. A copy of the mutants was resuspended in YEPD+20% glycerol, and stored in 200-µL aliquots at −70 °C. This stored copy served as a reference plate to prepare precultures for experiments. Mutants in in 96-well plates were served as inocula for screening of resistance to the antimicrobial activity of citral by incubating them for 24–48 h till growth appearance. The following reagents were mixed and filled with a volume of around 25 mL in Petri plates: 0.1% of DMSO, YEPD agar, in addition to the appropriate concentration of citral (MIC). Once the prepared mixture was solidified on petri plates, the replication from the liquid preculture mutants (kept at 30 °C for 24 h) was performed by using a 48-pin replicator (Figure 2). Each plate containing the mutants was repeated twice and incubated at 30 °C for 24–48 h.

### 2.4. In Silico Analyses

The Saccharomyces Genome Database (SGD) (http://www.yeastgenome.org/ (accessed on 12 January 2024)) and https://go.princeton.edu/ (accessed on 12 January 2024), the primary literature was consulted on an on-going basis to rationalize potential gene functions based on known protein partners, gene expression profiles, subcellular localization, synthetic lethal and other interactions, and any other relevant experimental observations. SGD GO Slim Mapper (https://www.yeastgenome.org/goSlimMapper (accessed on 12 January 2024)), gprofiler (https://biit.cs.ut.ee/gprofiler/gost (accessed on 13 January 2024)) and Gene Ontology (GO) Term Finder (https://www.yeastgenome.org/goTermFinder (accessed on 12 January 2024)) were used to set relations between deleted genes.

## 3. Results and Discussion

**Figure 3:**
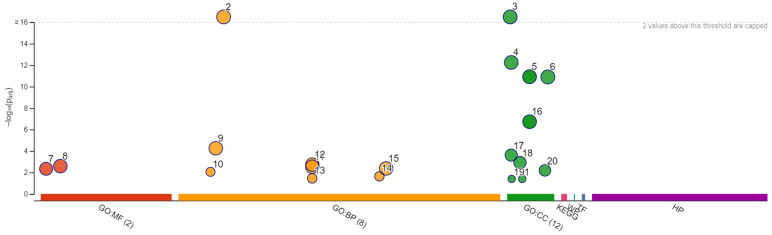
Manhattan plot grouping molecular functions (in red), biological processes (in orange), cellular components (in green). The x-axis displays functional words that have been color-coded and grouped by data source. The circle sizes correspond to the phrase size, so larger terms have larger circles, implying a greater number of genes. The y-axis displays the adjusted enrichment *p*-values in negative log10 scale. The meanings of the numbers are given in Table 1.

Growth of *S. cerevisiae* Σ1278b in the presence of different concentrations of citral was firstly carried out to assess the suitability of this strains as a model-strain to study the mechanisms of action of citral in yeast. Interestingly, the here-identified MIC of citral against Σ1278b (0.5 μL/mL) was identical to that already described in the literature against other yeast strains [17]. Subsequently, the complete library of Σ1278b haploid mutants deleted in 4019 non-essential genes was screened by using the MIC of citral determined on the wild-type strain. Systematic phenotypic and genetic analysis with the deletion mutant collection has proven highly useful because it allows thorough examination of mutations in each gene for specific phenotypes [37]. Thus, analyzing the resistance or sensitivity of the mutants toward the citral could lead to an understanding of the mechanisms or pathways behind citral action on yeast. Among the tested 4019 mutants, 509 proved to be resistant to citral (Table S1). To identify molecular functions, biological processes or cellular localizations common to the identified mutants, the list of the genes deleted in the resistant mutants was analyzed by using the Gene Ontology (GO) “TermFinder” and “Slim Mapper” as implemented in the Saccharomyces Genome Database website [39]. GO TermFinder is used to significantly identify highly enriched annotations among genes in major subclusters, while GO Slim Mapper maps granular annotations to high-level parent GO Slim terms. Figure 3 and Table 1 show the enrichment analysis findings and the significantly enriched GO categories, respectively.

In the “molecular function” main category, the sets ‘catalytic activity’ and ‘binding’ do not intersect. In “biological processes”, the sets ‘primary metabolic process’ and ‘filamentous growth’ intersect, as do the sets ‘growth of unicellular organisms’ and ‘organic substance metabolic process’. In “cellular component”, ‘mitochondrion’ and ‘organelle membrane’ intersect. This mean that these structures hold genes in common. As shown in the plot, the intracellular anatomical structure is separated from other biological processes, while the latter is significantly closed to the cytoplasm.

To assess the grade of comparability of the phenotypic profiles of individual deletion strains, and consequently the likely gene products in various structures, we progress deeply with each of the three structures: molecular function, cellular component, and biological process.

### 3.1. Molecular Function of the Identified Genes

A “molecular process” is defined as a process that can be performed by a single macromolecular machine, typically through direct physical interactions with other molecular entities. In this context, it refers to an action or activity carried out by a gene product (or a complex) (Table 2).

Among the 590 genes, 242 genes were grouped by catalytic activity with a *p* value of 0.00802, and four genes were grouped by thiosulfate sulfur transferase activity (*p* = 0.09019), where *YOR286W*, *YOR251C*, *YOR285W*, *YHR111W* have the two common functions. Concerning other child terms, 21 genes were grouped as transferase activity, and 38 genes as oxidoreductase activity (Table 2). Oxidoreductase activity is a child term of catalytic activity known as part of a large family of enzymes that catalyze oxidoreduction responses, such as electron transfer, proton/hydrogen extraction, hydride transfer, oxygen insertion, and the exchange of electrons between the donor and acceptor molecules.

### 3.2. Cellular Component of the Identified Genes

Deleted genes identified in citral resistant mutants are mostly located in the cytoplasm (1.15 × 10^−11^), and intracellular organelle (3.82 × 10^−10^). Mutants are related to each other in the following order: cellular anatomical entity (93.9%), intracellular anatomical structure (89.9%), organelle (80.8%), cytoplasm (80.5%), intracellular membrane-bound organelle and membrane-bound organelle (75.4%), membrane (37.1%), mitochondrion (26.3%), organelle membrane (26.1%), vacuole (11.2%), and vacuolar membrane (7.6%).

### 3.3. Biological Process of the Identified Genes

A “biological process” is defined as a specific target for which the organism is genetically programmed (SGD). Among 590 resistant mutants, the corresponding deleted genes are grouped as pseudohyphal growth (*p* = 0.02426), followed by regulation of pseudohyphal growth (*p* = 0.04274), and cell growth. Table 3 summarizes the map annotations of broad categories, using the GO Slim Mapper. Cluster frequency indicates that genes are close to each other under the same process, while “genome frequency” is the degree that a group of genes appear in a specific process when information is passed from parent to child in a cell. These two frequencies are associated in parallel, beginning with pseudohyphal growth, regulation of pseudohyphal growth, cell growth, growth of unicellular organism as a thread of attached cells, lipid metabolic process, ending with regulation of filamentous growth.

Table 4 shows the child terms of the biological process considered most significant. Mitochondrion organization (36 genes), mitotic cell cycle (32 genes,) and meiotic cell cycle (30 genes) were identified as important child terms of biological process. Other identified pathways pertained to lipid metabolic processes (52 of 590 genes), nucleobase-containing small molecule metabolic processes (20 of 590 genes), DNA recombination (19 of 590 genes), proteolysis involved in protein catabolic processes (8 of 590 genes) and DNA repair (25 of 590 genes). The knockout of these genes led to the survival of the corresponding mutants in the presence of citral, meaning that the indicated processes are potential targets of the antimicrobial action of citral. In particular, the perturbance of pseudohyphal growth and lipid metabolic processes seem to be linked to the resistance of the yeast mutants to citral. Other child terms of biological process are related to the growth of unicellular organisms as a thread of attached cells, filamentous growth of a population of unicellular organisms, mitochondrion organization, the mitotic cell cycle, the meiotic cell cycle, meaning that the perturbance of these processes led to the survival of the mutants. Among the different cellular processes, genes here identified and related to the cellular response to oxidative stress (mitochondrion organization) were further considered. Indeed, it has been previously suggested that citral increases the intracellular reactive oxygen species (ROS) [20].

### 3.4. Genes Related to the Cellular Response to Oxidative Stress

Oxidative stress is recognized as any process that alters a cell state or activity (such as movement, secretion, production of enzymes, gene expression) because of oxidative pressure, a state frequently resulting from exposure to elevated degrees of reactive oxygen species, for example superoxide anions, hydrogen peroxide (H_2_O_2_), and hydroxyl radicals (https://www.yeastgenome.org/ (accessed on 14 January 2024)). Table 5 lists genes related to cellular response to oxidative stress, and whose deletion confers Σ1278b resistance to citral. These genes are discussed individually in the following section.

*fRMsr* as well as *MsrA* and *MsrB* have antioxidant functions in yeast and regulate oxidative stress. *MsrA* and *MsrB* were discovered to regulate mitochondrial activities in yeast by the cellular mechanism for methionine sulfoxide reduction [42]. These enzymes, however, had differential effects on mitochondrial activity, with *MsrA* largely found in the cytoplasm and *MsrB* in mitochondria. Kaya et al. [63] reported the sensitivity of Δ*ykl069w* to H_2_O_2_, leading to cell death.

*AFG1* and *PUF3* have a physical interaction whereas Puf3p (citral enables this function) is expected to play a function in translation control under oxidative stress conditions, with its role in repression of protein synthesis linked to mitochondrially active genes [64]. The minimal phenotypic manifestation in Δ*afg1* cells suggests that yeast mitochondria are resistant to a large degree of mitochondrial depolarization and ROS [41].

As mentioned by Gebert et al. [65], the knockout of *GRE3* leads to the increase of the pentose phosphate pathway, through the reactions that detoxify ROS with glutathione and thioredoxin. In addition, Δ*gre3* cells experienced a significant increase in H_2_O_2_ and O_2_ concentrations during chronological ageing in comparison to wild-type yeast cells.

*TIM18* is located in mitochondria and is part of the *TIM22* mitochondrial import inner membrane insertion complex [65]. Nakamura et al. [52] showed that yeast’s response to apoptotic stress and other type of stresses is affected by *TIM18* deficiency, and that this gene is considered a downstream component of ROS formation. In addition, the Δ*tim18* mutant was resistant to some stresses that stimulate apoptosis, such as hydrogen peroxide.

Δ*ynl080c* showed a high sensitivity to oxidative stress [52]. Higgins et al. [54], reported that yeast’s tolerance to oxidative stress depends on *EOS1*.

Δ*gpx1* showed sensitivity to all stresses tested, such as H_2_O_2_, and this gene showed importance for yeast growth under oxidative stress [61]. However, it has also been suggested that *MPR1* probably compensates for the role of *GPX1* in the deleted mutant, by reducing intracellular ROS accumulation.

As reported by Lee et al. [62], deletion of Δ*mcr1* proved resistant to an antimicrobial hexapeptide.

*SRX1* enhances oxidative stress resistance by reducing cysteine-sulfinic acid groups in the peroxiredoxin Tsa1p. Srx1p and Prx1p have a physical interaction, where *PRX1* is a mitochondrial peroxiredoxin with thioredoxin peroxidase activity, induced during oxidative stress and reactivation requires Trr2p and glutathione [58].

Costa et al. [66] and MacDiarmid et al. [49] found an increased sensitivity of Δ*trr2* to H_2_O_2_, suggesting the important role of the mitochondrial thioredoxin reductase in protection against oxidative stress in *S. cerevisiae*. The mitochondrial thioredoxin proteins thioredoxin (Trx3p) and thioredoxin reductase (Trr2p) have been linked to oxidative stress defense during respiratory metabolism [59].

TSA1 codifies for a peroxiredoxin that reduces hydrogen peroxide and organic hydroperoxides by using electrons from the thioredoxin/thioredoxin reductase pathway. The Δ*tsa1* mutant was proven to be sensitive to H_2_O_2_ and ethanol [48,66,67,68,69]. Another study showed that the Δ*trr1* increased oxidant tolerance in a Δ*tsa1* mutant. However, it was confirmed that *TRR1* significantly reduced resistance to hydrogen peroxide, either alone or in combination with Δ*tsa1*. When two conserved cysteines create an intramolecular disulfide bond, they exist in either a reduced form (thioredoxin-(SH)2) or an oxidized form (thioredoxin-S2). Thioredoxins transfer electrons from their active center dithiol to disulfide bonds in proteins (Protein-S2), which are then reduced to dithiols (Protein-(SH)2). *TRR1* directly reduces the resultant oxidized thioredoxin disulfide with electrons provided by NADPH. Thioredoxin, thioredoxin reductase, and NADPH comprise the thioredoxin reduction system. Oxidized glutaredoxins, on the other hand, are reduced using electrons given by NADPH by the tripeptide glutathione (gamma-Glu-Cys-Gly, or GSH). Thus, glutaredoxin, glutathione, glutathione reductase, and NADPH comprise the glutathione/glutaredoxin system [70]. The expression of *TRR1* is regulated by the transcription factors Yap1p and Skn7p in response to H_2_O_2_ [71].

Park et al. [47] demonstrated that *HSP31* is involved in the protection against reactive oxygen species (ROS) and that oxidative stress induces its expression in a Yap1p-dependent manner. This could be explained by the possibility that the induction of the Yap1p gene is dependent on the presence of functional Yap1p or Hsp31p synthesis, which is regulated independently of Yap1p by the level of intracellular ROS. *SOD1* and *HSP31* may complement each other [45], and it was demonstrated that oxidative stress induces *HSP31* expression. Several studies [72,73,74] have found that Yap1p is involved in the upregulation of oxidative stress response genes after exposure to reactive oxygen species. Yap1p binding sites are found in the promoters of most genes that are strongly induced in response to ROS, including cytosolic catalase (*CTT1*), cytosolic superoxide dismutase (*SOD1*), gamma glutamylcysteine synthetase (*GSH1*), thioredoxin 2 (*TRX2*), and thioredoxin reductase (*TRR1*) [50,75]. The formation of an intramolecular disulfide in one or more cysteine-rich domains is thought to trigger Yap1p redistribution from the cytoplasm to the nucleus [76].

## 4. Conclusions

In conclusion, based on the above results, the identified 509 mutants resistant to citral were subdivided into categories depending on GO biological process, molecular function, and cellular component of the deleted genes. The categories “mitochondrion organization” (36 genes), “mitotic cell cycle” (32 genes), and “meiotic cell cycle” (30 genes) comprised the highest number of mutants. Furthermore, many of the identified genes were related to pathways responsible for oxidative stress, such as ROS scavenging, and could be involved in different mechanisms. In particular, the antimicrobial activity of citral may concern the induction of Yap1p, the increase in the level of intracellular ROS, the decrease of the pentose phosphate pathway, or the triggering of other proteins involved in the membrane-embedded core of the *TIM22* complex. Thus, it is conceivable that citral is targeting the antioxidant response of the cell. Also, citral might have a role in stimulating the thioredoxin system, by mitochondrial peroxiredoxin (*PRX1*), and the mitochondrial thioredoxin proteins thioredoxin (Trx3p) and thioredoxin reductase (Trr2p), that are linked to oxidative stress defense. Also, citral is probably increasing the role of *TRR1* (thioredoxin reductase). By analyzing the Σ1278b *S. cerevisiae* mutant-gene bank, we contributed for first time to the molecular biology knowledge of the mechanisms of action of antimicrobial molecules, with particular emphasis on the resistance produced by natural or induced mutations, and investigated effective methods for preventing unwanted microbial growth, especially in food matrices, promoting the possibility that citral may be used safely and efficiently, and helping to understand which mechanisms underlie the development of tolerance or resistance of microorganisms to citral.

## Figures and Tables

**Table 1:** Term name of cellular components (CC), biological processes (BP) and molecular functions (MF).

ID	Source	Term ID	Term Name	P_adj_ (Query_1)
1	GO:CC	GO:0031907	Microbody lumen	4.029 × 10^−2^
2	GO:BP	GO:0009987	Cellular process	2.188 × 10^−18^
3	GO:CC	GO:0005622	Intracellular anatomical structure	8.594 × 10^−17^
4	GO:CC	GO:0005737	Cytoplasm	5.573 × 10^−13^
5	GO:CC	GO:0043226	Organelle	1.212 × 10^−11^
6	GO:CC	GO:0110165	Cellular anatomical entity	1.248 × 10^−11^
7	GO:MF	GO:0003824	Catalytic activity	4.577 × 10^−3^
8	GO:MF	GO:0005488	Binding	2.577 × 10^−3^
9	GO:BP	GO:0008152	Metabolic process	5.582 × 10^−5^
10	GO:BP	GO:0007124	Pseudohyphal growth	8.798 × 10^−3^
11	GO:BP	GO:0044237	Cellular metabolic process	2.940 × 10^−3^
12	GO:BP	GO:0044238	Primary metabolic process	1.856 × 10^−3^
13	GO:BP	GO:0044182	filamentous growth of a population of unicellular organisms.	3.599 × 10^−2^
14	GO:BP	GO:0070783	growth of unicellular organism as a thread of attached cells	2.302 × 10^−2^
15	GO:BP	GO:0071704	Organic substance metabolic process	4.253 × 10^−3^
16	GO:CC	GO:0043231	Intracellular membrane-bounded organelle	1.856 × 10^−7^
17	GO:CC	GO:0005739	mitochondrion	2.429 × 10^−4^
18	GO:CC	GO:0031090	Organelle membrane	1.198 × 10^−3^
19	GO:CC	GO:0005782	Peroxisomal matrix	4.029 × 10^−2^
20	GO:CC	GO:0098588	Bounding membrane of organelle	6.426 × 10^−3^

**Table 2:** Important child terms of MF.

GO Ontology Term	Genes	GO ID
Oxidoreductase activity	38 of 590 genes, 6.44%	GO:0016491
Transferase activity	21 of 590 genes, 3.56%	GO:0016740
Kinase activity	18 of 590 genes, 3.05%	GO:0016301

**Table 3:** Classification of deleted genes according to biological process.

Gene Ontology Term	Cluster Frequency	Genome Frequency	Corrected *p*-Value	FDR	FALSE Positives	Genes Annotated to the Term
pseudohyphal growth	18 of 590 genes, 3.1%	73 of 7166 genes, 1.0%	0.02426	0.00%	0.00	YOR032C, YKL149C, YNL068C, YHR111W, YNL076W, YKL185W, YOR315W, YNL196C, YLR353W, YER020W, YNL142W, YJR094C, YOR030W, YNL098C, YHR084W, YLR362W, YDR480W, YDR477W
regulation of pseudohyphal growth	9 of 590 genes, 1.5%	22 of 7166 genes, 0.3%	0.04274	2.00%	0.04	YNL068C, YHR111W, YNL076W, YNL098C, YHR084W, YKL185W, YNL196C, YDR477W, YDR480W
cell growth	19 of 590 genes, 3.2%	85 of 7166 genes, 1.2%	0.06294	1.33%	0.04	YOR032C, YKL149C, YNL068C, YHR111W, YNL076W, YKL185W, YOR315W, YNL196C, YJL201W, YLR353W, YER020W, YNL142W, YJR094C, YOR030W, YNL098C, YHR084W, YLR362W, YDR480W, YDR477W
growth of unicellular organism as a thread of attached cells	21 of 590 genes, 3.6%	100 of 7166 genes, 1.4%	0.06823	2.50%	0.10	YOR032C, YKL149C, YNL068C, YHR111W, YNL076W, YKL185W, YOR315W, YNL196C, YOR212W, YLR353W, YER020W, YNL142W, YJR094C, YNL097C, YOR030W, YNL098C, YHR084W, YLR362W, YOR080W, YDR480W, YDR477W
lipid metabolic process	52 of 590 genes, 8.8%	365 of 7166 genes, 5.1%	0.07262	2.00%	0.10	YNL111C, YDR503C, YOR317W, YEL020C, YOR100C, YOR245C, YOR022C, YNL156C, YNL045W, YJL062W, YNL202W, YOR025W, YOR237W, YJR066W, YHR133C, YOR126C, YOR365C, YOR221C, YJR019C, YOR298W, YOR175C, YOR093C, YKL094W, YJL187C, YLR404W, YKL140W, YKL055C, YOR311C, YJL100W, YJL134W, YOR084W, YOR086C, YHR100C, YNL169C, YOR059C, YKL150W, YER024W, YNL123W, YNL054W, YLR386W, YNL231C, YOR180C, YOR081C, YHR004C, YNL128W, YOR196C, YER005W, YKL188C, YJL083W, YLR450W, YER019W, YHL032C
regulation of filamentous growth	12 of 590 genes, 2.0%	40 of 7166 genes, 0.6%	0.07885	1.67%	0.10	YNL068C, YNL142W, YHR111W, YNL097C, YNL076W, YNL098C, YHR084W, YKL185W, YOR315W, YNL196C, YDR477W, YDR480W

**Table 4:** Important child terms of biological process.

GO Ontology Term	Genes	GO ID
mitochondrion organization	36 of 590 genes, 6.10%	GO:0007005
mitotic cell cycle	32 of 590 genes, 5.42%	GO:0000278
meiotic cell cycle	30 of 590 genes, 5.08%	GO:0051321
transmembrane transport	26 of 590 genes, 4.41%	GO:0055085
organelle fission	22 of 590 genes, 3.73%	GO:0048285
cell wall organization or biogenesis	21 of 590 genes, 3.56%	GO:0071554
cellular ion homeostasis	20 of 590 genes, 3.39%	GO:0006873
nucleobase-containing small molecule metabolic process	20 of 590 genes, 3.39%	GO:0055086
chromatin organization	19 of 590 genes, 3.22%	GO:0006325
nucleobase-containing small molecule metabolic process	20 of 590 genes, 3.39%	GO:0055086
DNA recombination	19 of 590 genes, 3.22%	GO:0006310
proteolysis involved in protein catabolic process	8 of 590 genes, 3.05%	GO:0051603
DNA repair	25 of 590 genes, 4.24%	GO:0006281

**Table 5:** Summary of previous studies related to deleted genes in selected resistant mutants. Chemicals such as hydrogen peroxide, paraquat, and menadione were used in prior studies to study their resistance or sensitivity in the yeast.

Deleted Gene	Chemical	References	Chemical	References
*GCY1* *(YOR120W)*				No references available
*AFG1* *(YEL052W)*			hydrogen peroxide	oxidative stress resistance: decreased [40,41]
*YKL069W* *fRMsr*, *YKG9*	Hydrogen peroxide	Oxidative stress resistance increased [40]	Hydrogen peroxide	oxidative stress resistance: decreased [42]
*SRX1/YKL086W*			Paraquat hydrogen peroxide	oxidative stress resistance: decreased [43,44]
*GRE3/YHR104W*				
*LTV1/YKL143W (YKL2)*	paraquat	oxidative stress resistance: increased. [44]		
*HSP31/YDR533C*			lipid hydroperoxide hydrogen peroxide tert-butyl hydroperoxide 1,1′-azobis(N,N-dimethylformamide)	oxidative stress resistance decreased [45,46]
*TSA1/YML028W*			hydrogen peroxide tert-butyl hydroperoxide 1,1′-azobis (N,N-dimethylformamide Rose Bengal paraquat	oxidative stress resistance decreased [47,48,49,50]
*TIM18/YOR297C*	Hydrogen peroxide	oxidative stress resistance increased [51]		
*EOS1/YNL080C*	paraquat	oxidative stress resistance increased [44]	cumene hydroperoxide hydrogen peroxide linoleic acid hydroperoxide	oxidative stress resistance: decreased [52,53,54]
*GRR1/YJR090C*			Paraquat hydrogen peroxide tert-butyl hydroperoxide dioxygen	oxidative stress resistance: decreased [53,55,56]
*UBA4/YHR111W*	Hydrogen peroxide	oxidative stress resistance increased [40]	1,1′-azobis(N,N-dimethylformamide)	oxidative stress resistance: decreased [57]
*TRR2/YHR106W*			cadmium (2+) hydrogen peroxide	oxidative stress resistance: decreased [48,58,59]
*TOR1/YJR066W*	Hydrogen peroxide	oxidative stress resistance increased [60]		
*GPX1/YKL026C*			Hydrogen peroxide	oxidative stress resistance: decreased [48,61]
*MCR1/YKL150W*	Menadione Hydrogen peroxide	oxidative stress resistance increased [40,62]	Hydrogen peroxide menadione	oxidative stress resistance decreased [62]

## Data Availability

The original contributions presented in the study are included in the article/Supplementary Materials, further inquiries can be directed to the corresponding author.

## Supplementary Materials

The following supporting information can be downloaded at: https://www.mdpi.com/article/10.3390/foods13101457/s1, Table S1.
